# Imerslund-Gräsbeck Syndrome in an Infant with a Novel Intronic Variant in the *AMN* Gene: A Case Report

**DOI:** 10.3390/ijms20030527

**Published:** 2019-01-27

**Authors:** Alessandra Pacitto, Paolo Prontera, Gabriela Stangoni, Maurizio Stefanelli, Stefania Ceppi, Carla Cerri, Grazia Gurdo, Annalisa Mencarelli, Susanna Esposito

**Affiliations:** 1 Pediatric Clinic, Department of Surgical and Biomedical Sciences, Università degli Studi di Perugia, Piazza Menghini 1, 06129 Perugia, Italy; alessandra-pacitto@libero.it (A.P.); maurizio.stefanelli@ospedale.perugia.it (M.S.); dh.pediatria.silv@ospedale.perugia.it (S.C.); almiomedico@gmail.com (A.M.); 2 Medical Genetics Unit, Santa Maria della Misericordia Hospital, Piazza Menghini 1, 06129 Perugia, Italy; paolo.prontera@ospedale.perugia.it (P.P.); gabriela.stangoni@ospedale.perugia.it (G.S.); 3 Pediatric Oncohematology Unit, Santa Maria della Misericordia Hospital, Piazza Menghini 1, 06129 Perugia, Italy; Carla.cerri@ospedale.perugia.it (C.C.); grazia.gurdo@ospedale.perugia.it (G.G.)

**Keywords:** Imerslund-Gräsbesck syndrome, cobalamin deficiency, macrocytic anemia, proteinuria, amnionless (AMN), cubilin (CUBN)

## Abstract

Imerslund-Gräsbeck syndrome (IGS) is a rare autosomal recessive disorder clinically characterized by megaloblastic anemia, benign mild proteinuria, and other nonspecific symptoms. Several pathogenetic variants in the amnionless (*AMN*) or cubilin (*CUBN*) genes have been described in IGS. We describe a case of IGS with urinary tract infection and mild but persistent proteinuria at onset in an 11-month-old female child. With the appearance of macrocytic anemia, aphthous stomatitis, and neurological signs, IGS was clinically suspected, and vitamin B12 parenteral therapy was started. Sequence analysis showed the presence of a novel intronic variant c.513+5G>A of *AMN*, never before described in the literature, that was in compound heterozygosity with the known pathogenetic variant c.1006+34_1007-31del. Analysis extension to the parents revealed the presence of variant c.1006+34_1007-31 in the father and c.513+5G>A in the mother. In the present case with IGS, the novel intronic variant of *AMN* was identified in “*trans*” with a known pathogenic variant (c.1006-31 del) and the new variant was interpreted to be pathogenetic since it was not found in the public database of polymorphisms and because it was predicted to alter a donor splicing site. Our case underlines the relevance in detecting certain subtle symptoms, such as mild but persistent proteinuria associated with megaloblastic anemia, to reach a correct diagnosis of a rare but treatable disorder.

## 1. Introduction

Imerslund-Gräsbeck syndrome (IGS), also known as “Juvenile Megaloblastic anemia type 1” or “Selective vitamin B12 malabsorption with proteinuria”, is a rare genetic disorder inherited through an autosomal recessive pathway [1].

IGS impairs the absorption of vitamin B12 and in some cases also the reabsorption of proteins in the kidney [2,3]. The molecular basis of selective malabsorption of vitamin B12 and proteinuria are linked to mutations of cubilin (*CUBN*) or amnionless genes (*AMN*). The two proteins encoded by these genes are part of the receptor for the complex vitamin B12-intrinsic factor (IF) in the ileum and of the receptor that mediates the reuptake of proteins in primary urine [4,5]. The gene map loci are 10p12.1 for *CUBN* and 14q32 for *AMN*. Various mutations have been described. *CUBN* and *AMN* biallelic pathogenic variants were found in Norwegian and Finnish families, respectively, while in Mediterranean IGS cases, *CUBN* and *AMN* variants have both been reported [4,5]. The disease is clinically diagnosed and is confirmed by genetic analysis. Treatment consists of lifelong vitamin B12 parental therapy and, when started early, guarantees an excellent prognosis, preventing psychomotor delay in children. This case report describes a new intronic variant (c.513+5G>A) located in “*trans*” with an already-known pathogenetic variant (c.1006-31del) in the *AMN* gene in an infant with IGS.

## 2. Case Presentation

A 11-month-old female Italian infant was admitted to our hospital because of three days of fever, vomiting, and worsening of her general conditions. Physical examination revealed a febrile and pale child with a normal neurological status. Axillary temperature was 40 °C, and the refill time was 2 s. There were no signs of upper or lower airway infections or meningeal involvement. Her weight was 10 kg (50th percentile), her length was 74 cm (50th percentile) and her head circumference was 44 cm (45th percentile). Her growth showed a linear trend from birth, and she reached psychomotor milestones regularly. She was born at term, and she was breastfed until six months. She followed a various and complete diet. There was no history of consanguinity, blood disorders or kidney diseases.

The initial work-up showed leucocytosis (white blood cells, 11,620 × 10^3^/µL); normochromic normocytic anemia with hemoglobin values under 2 standard deviation (SD) for age (Hb, 8.6 g/dL); MCV and MCH, normal for her age (MCV 75.9 fl; MCH 24.6 pg); and a mild increase in C reactive protein (CRP, 2 mg/dL). Kidney function was normal (creatinine, 0.21 mg/dL; azotaemia, 20 mg/dL), whereas urine was turbid with 10^4^ leucocytes (UCF), a low-grade proteinuria measured on an extemporaneous sample (100 mg/dL), and urine culture resulted positive for *Escherichia coli*. Diagnosis of urinary tract infection was made, and intravenous therapy with ceftazidime was started. After eight days, the infant was discharged from our hospital with normal blood exams and anemia was interpreted as a transient finding during an acute infective process.

The child missed her first follow-up visit, and 17 months later, when she was 28 months old, during an occasional visit a mild-moderate proteinuria with a mixed tubular-glomerular pattern was detected (187 mg/dL). On a 24-h urine sample, an elevated proteinuria/creatininuria ratio was found (patient’s ratio: 2, normal values <0.2) with proteinuria (60 mg/dL) and creatininuria (30 mg/dL). A blood exam revealed macrocytosis (MCV 98.5 fl) in the absence of anemia (Hb 12.4 g/dL). Renal ultrasound showed no anomalies of kidneys or the urinary tract. However, she presented oral aphthous ulcers, vulvar hyperaemia, and abnormal movements of buccal rhyme. We finally dosed peripheral blood levels of folate and vitamin B12, finding a severe vitamin B12 deficiency (0.1 pg/mL; normal range values 180–914 pg/mL). Vitamin B12 was analyzed using Electrochemiluminescence ImmunoAssay (Roche Diagnostics GmbH, Mannheim, Germany) and the low value was identified using dilution methods. The combination between severe B12 deficiency and proteinuria was strongly suggestive of IGS. Table 1 summarizes the clinical and laboratory findings at admission and during follow-up: normal values for age are shown; we decided to replace vitamin B12 intramuscularly (IM). A first administration of cyanocobalamin (200 mcg/day) IM for two consecutive days was given, followed by administration of 100 mcg/day for seven days; the maintenance dose consisted of 100 mcg a week for one month (four doses overall) followed by 100 mcg administered monthly. The first vitamin B12 dosage was made after one week and after four months from the beginning of the therapy. Vitamin B12 levels increased rapidly: one week later, vitamin B12 values were normal (622 pg/mL), anemia resolved (Hb 12.8 g/dL; MCV 94.1 fl; MCH 31.7 pg), while mild proteinuria persisted (70 mg/dL). At the 4-month follow-up of therapy, vitamin B12 levels were still in the normal rage (169 pg/mL), hemoglobin was normal (Hb 13.5 g/dL), urine extemporaneous proteinuria levels were 60 mg/dL, and there was no macrocytosis (MCV 75.8 fl).

Considering the clinical and laboratory findings, i.e., macrocytosis and persistent proteinuria with a severe lack of vitamin B12, the child was assessed for IGS. DNA was extracted from peripheral blood of the patient, and sequencing of the coding regions and of the exon–intronic junction of the *AMN* gene was performed. The variants identified in the proband were analyzed in the parents through Sanger sequencing. Sequence analysis showed the presence of a novel intronic variant c.513+5G>A of *AMN*, never before described in the literature, that was in compound heterozygosity with the known pathogenetic variant c.1006+34_1007-31del. Indeed, analysis extension to the parents revealed the presence of variant c.1006+34_1007-31 in the father and c.513+5G>A in the mother. 

Clinical information and blood samples were obtained after approval from the Ethics Committee (PED-2018-18, 15 September, 2018) of the Umbria Region with signed informed consent by both parents. Parents also signed the consent for the publication of this case report.

## 3. Discussion

This case describes a female child with IGS who presented at onset urinary tract infection associated with mild but persistent benign proteinuria in the absence of kidney damage and who later showed megaloblastic anemia, in whom sequencing of the entire coding region and flanking intronic regions of the *AMN* gene revealed compound heterozygous variants with a new intronic variant never described in the literature (c.513+5G>A).

The age of onset in our case is in line with the literature because first symptoms usually appear from four months of age to early childhood [1,6]. Although proteinuria can be absent, the association of megaloblastic anemia and benign mild proteinuria, resistant to parenteral vitamin B12 therapy, should always suggest IGS. In our case, as described in the literature [7], there was no kidney damage, creatinine and azotaemia were always in range, and kidney function remained intact during the whole follow-up period. With the clinical suspicion of IGS, we rapidly initiated vitamin B12 treatment, starting with an attack dose, and performed monthly injections, gaining a rapid resolution of anemia and other symptoms. The persistence of proteinuria was an ex-adjuvantibus criterion for diagnosis before the availability of genetic exams. 

Sequencing of the *AMN* gene identified two allelic variants c.513+5G>A and c.1006-31 del, confirming the clinical diagnosis of IGS in our patient. In particular, we found a new intronic variant c.513+5G>A, which segregated with the mother, in “*trans*” with a pathogenic variant, which segregated with the father (c.1006-31 del). Although this new variant (c.513+5G>A) has never been reported in the literature in association with IGS, it has also not been found in the general population as a polymorphism (GnomAD, ExAC, 1000G). Moreover, this new variant is located near a splicing donor site and the bioinformatic tool “Human Splicing Finder” predicts that it alters the splicing process. Taken together, these observations strongly suggest a pathogenetic role for this new *AMN* variant.

## 4. Conclusions

In the present case report, a novel intronic variant of *AMN* (c.513+5G>A) was identified in “*trans*” with a known pathogenic variant (c.1006-31 del) in a female child with a phenotype suggestive of IGS. The new variant was interpreted to be pathogenetic since it was not found in the public database of polymorphisms (GomAD, ExAC, 1000G; available at http://exac.broadinstitute.org/Accessed on 22 December, 2018) and because it was predicted to alter a donor splicing site. This case also underlines the relevance in detecting certain subtle symptoms, such as mild proteinuria associated with megaloblastic anemia, to reach a correct diagnosis of a rare but treatable disorder.

## Figures and Tables

**Table 1:** Clinical and laboratory findings at admission and during the follow-up.

Finding	Admission (11-Months-Old)	After 7 Months (I Follow-Up)	After 12 Months (II Follow-Up)	After 13 Months (Post-Treatment I with Vitamin B12)	After 18 Months (Post-Treatment II with Vitamin B12)
**Symptoms/Signs**					
	Urinary tract infection due to *Escherichia coli*	No symptom or sign	Oral aphthosis, vulvar hyperemia and abnormal movements of buccal rhyme	No symptom or sign	No symptom or sign
**Blood Exams**					
Leucocyte, cells/µL (6000–17,500)	11,620	7780	11,620	9740	10,031
Erythrocytes, cells/µL (3,700,000–4,900,000)	3,490,000	3,370,000	3,860,000	4,040,000	5,120,000
Hemoglobin, g/dL (10.5–13)	8.6	12.4	12.4	12.8	13.5
Hematocrit, % (33–38)	30.5	33.2	36.5	38	38.8
MCV, fl (70–84)	75.9	98.5	94.8	94.1	75.8
MCH, pg (23–30)	24.6	34.1	32.1	31.7	24.6
Azotemia, mg/dL (25–40)	20	28	32	31	23
Creatinine, mg/dL (0.3–0.7)	0.21	0.28	0.29	0.33	0.24
Vitamin B12, pg/mL (180–914)	Not checked	Not checked	0.1	622	169
Folic acid, ng/mL (2–9)	Not checked	Not checked	>23.7	Not checked	Not checked
**Urine Exam**					
Single-sample urine-proteinuria	100 mg/dL	160 mg/dL	187.50 mg/dL	70 mg/dL	60 mg/dL
24 h urine proteinuria	Not checked	60 mg/dL	Not checked	Not checked	Not checked
24 h urine creatininuria	Not checked	30 mg/dL	Not checked	Not checked	Not checked
Proteinuria/Creatininuria ratio	Not checked	2	Not checked	Not checked	Not checked

Age-specific reference intervals in parenthesis.
